# Supplementary material for: Canonical androgen response element motifs are tumor suppressive regulatory elements in the prostate
Source: Nat Commun. 2024 Dec 13;15:10675. doi: 10.1038/s41467-024-53734-z (PMC11645413; doi:10.1038/s41467-024-53734-z)
Supplement: Supplementary file 1 — Supplementary Information [file 41467_2024_53734_MOESM1_ESM.pdf]

Supplementary Information

List:

Supplementary Figure 1-12

Supplementary Table 1 ARE repression score with clinical characteristics in localized prostate cancer patient cohort.

Supplementary Table 2 Univariable and multivariable Cox regression analyses of ARE repression score.

Supplementary Table 3 ARE activation score with clinical characteristics in localized prostate cancer patient cohort.

Supplementary Table 4 Univariable and multivariable Cox regression analyses of ARE activation score.

Supplementary Table 5 Oligos or DNA sequences used in this study.

Supplementary Table 6 Antibodies used in this study.

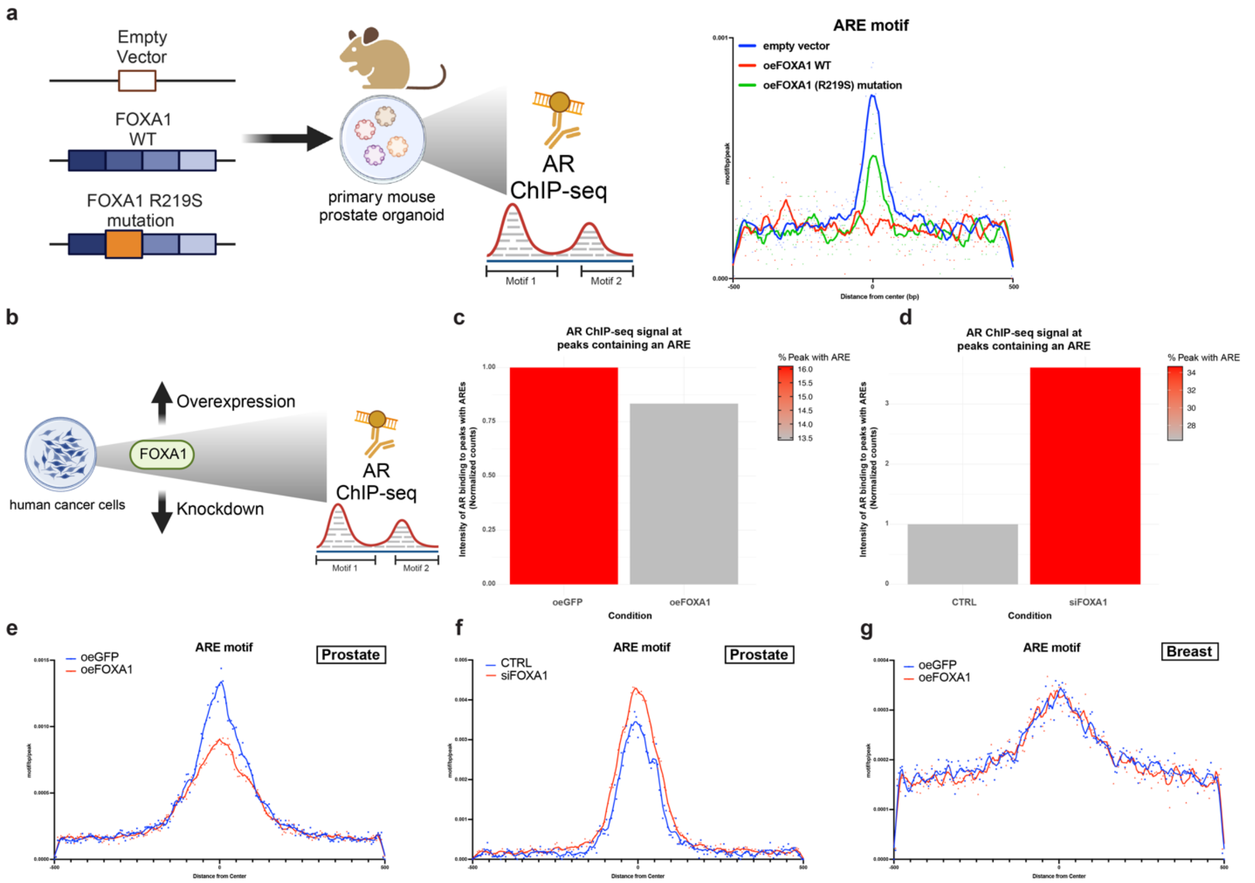

Supplementary Fig. 1 ARE motifs are de-enriched in multiple prostate models.
a, Schematic of motif enrichment analysis from AR ChIP-seq in mouse prostate organoid models with FOXA1 overexpression (GSE128867). Histograms depicting the normalized intensity of AR binding to peaks containing an ARE within a 500-bp window around the peak centers in cells that FOXA1 wild-type (WT) overexpression against an empty vector control and compared these with the FOXA1 R219S mutation, a loss-of-function mutation known to abrogate FOXA1 DNA binding. **b,** Schematic of motif enrichment analysis from AR ChIP-seq in androgen-responsive PCa cell models, specifically with FOXA1 overexpression and knockdown. **c,** Intensity of AR binding in peaks (E-MTAB-1749) with an ARE in androgen responsive LNCaP cells with overexpression of FOXA1 or GFP control conditions. **d,** Intensity of AR binding in peaks (GSE30623) with an ARE in androgen responsive LNCaP-1F5 cells with depletion of FOXA1 (siFOXA1) or control conditions. **e-f,** Histograms depicting the normalized intensity of AR binding to peaks containing an ARE within a 500-bp window around the peak centers in androgen-responsive LNCaP and LNCaP-1F5 cells, respectively. The analysis compares conditions of FOXA1 overexpression and GFP control based on data from E-MTAB-1749, as well as FOXA1 depletion and control conditions in LNCaP-1F5 cells from GSE30623. **g,** Histograms depicting the normalized intensity of AR binding to peaks containing an ARE within a 500-bp window around the peak centers in breast lineage-based AR-positive MDA-MB-453 cells. Input files were from FOXA1 overexpression condition (red line) and GFP control condition (blue) AR ChIP-seq peaks. Source data are provided as a Source Data file. **a-b**, Created with BioRender.com.

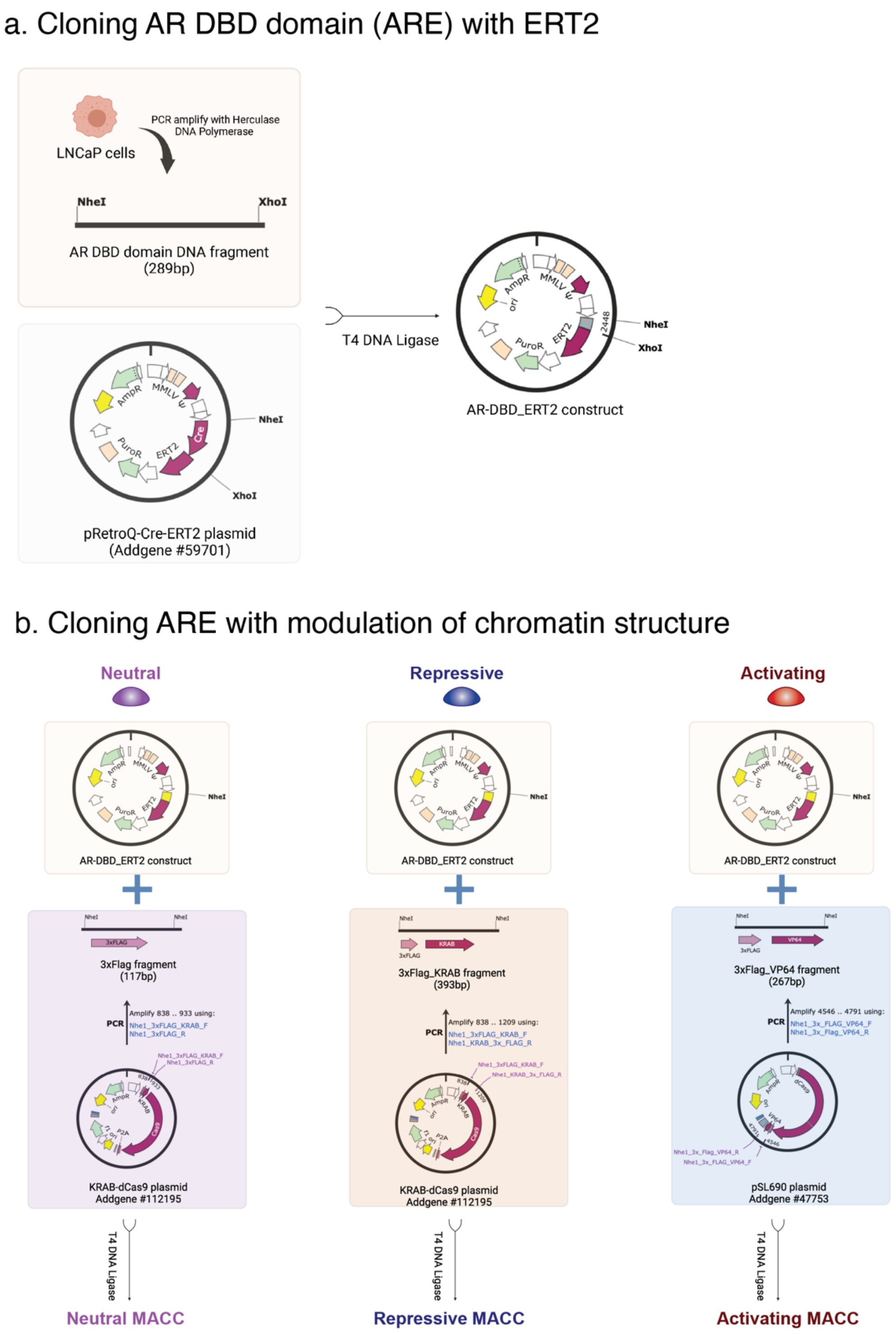

Supplementary Fig. 2 Cloning strategy of MACC constructs.
a, Cloning AR DBD domain (ARE) with ERT2 to achieve tamoxifen-inducible ability. **b,** Cloning ARE with modulation of chromatin structure. **a**, Created with BioRender.com.

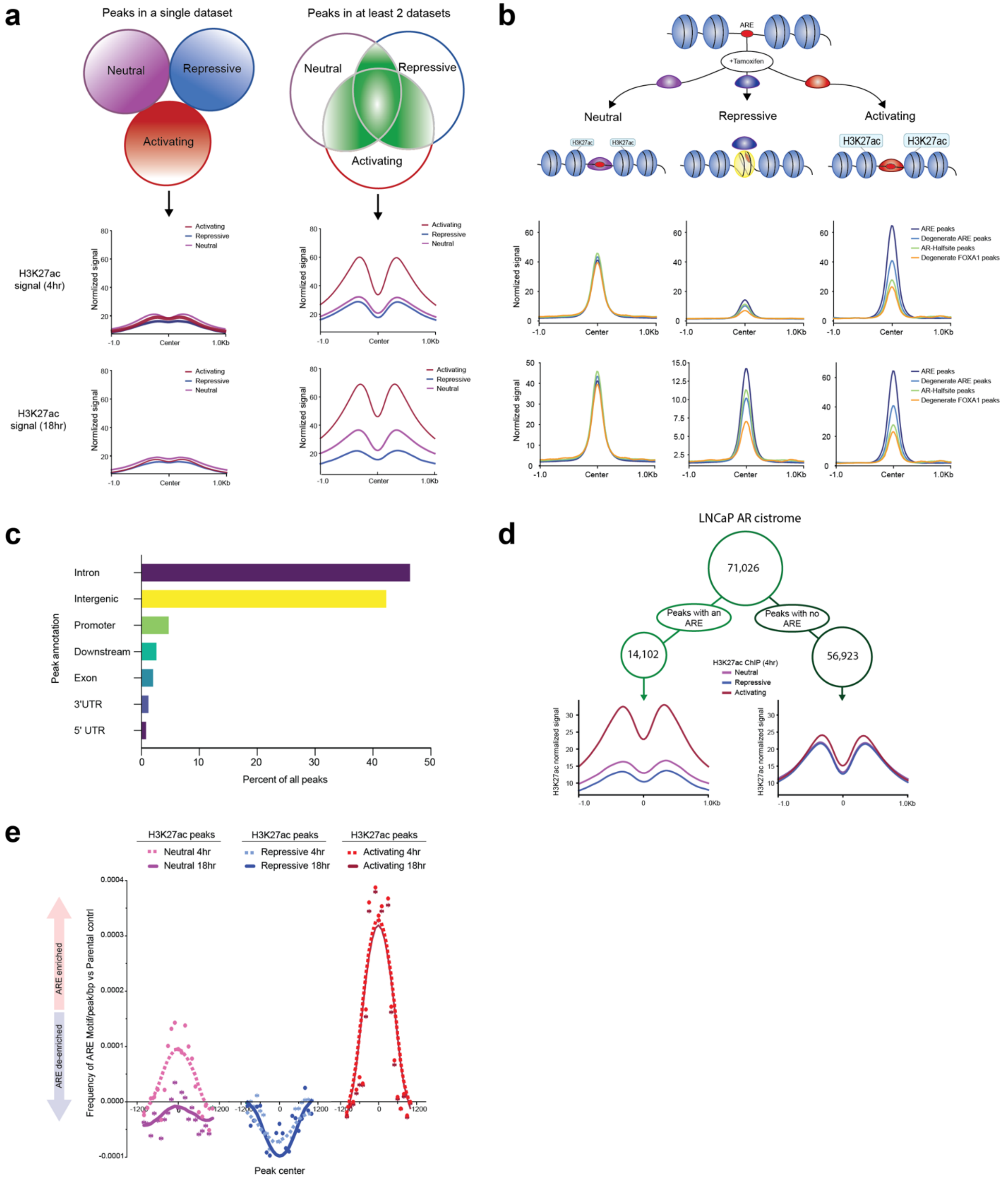

Supplementary Fig. 3 Generation and validation of consensus MACC ChIP-seq peaks and association with H3K27ac activity.

a, H3K27ac activity of all MACC peaks in different intersections (Neutral, Repressive, and Activating) at 4hr and 18hr. **b,** Peak activity (H3K27ac) distribution of the ARE peaks, degenerate ARE peaks, AR half-site peaks, and degenerate FOXA1 peaks in MACC settings (Top-similar

scale, Bottom-Free scale). **c,** Annotation of the MACC peaks by genomic localization as a percentage of all peaks. **d,** Peak activity distribution of LNCaP AR peaks with or without ARE in MACC H3K27ac (4hr) settings. **e,** Changes in ARE motif frequency over parental control of the MACC H3K27ac) peaks (4hr and 18hr .

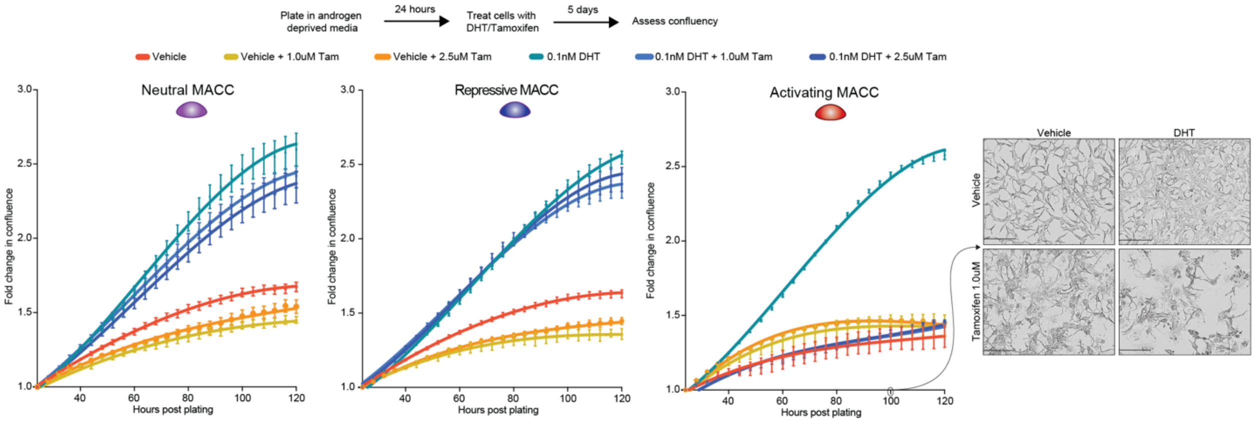

Supplementary Fig. 4 Growth of LNCaP MACC lines with vehicle or tamoxifen and androgen deprived or DHT stimulated condition.

The growth of LNCaP MACC lines in 2D culture was assessed after androgen deprivation and DHT stimulation, with the addition of either vehicle or tamoxifen, n=3. Cell growth was monitored by live-cell imaging throughout the 5-day treatment period to observe any changes in proliferation rates. Curves represent confluence; representative brightfield images on right. Experiments were conducted at least three times with consistent results. Source data are provided as a Source Data file.

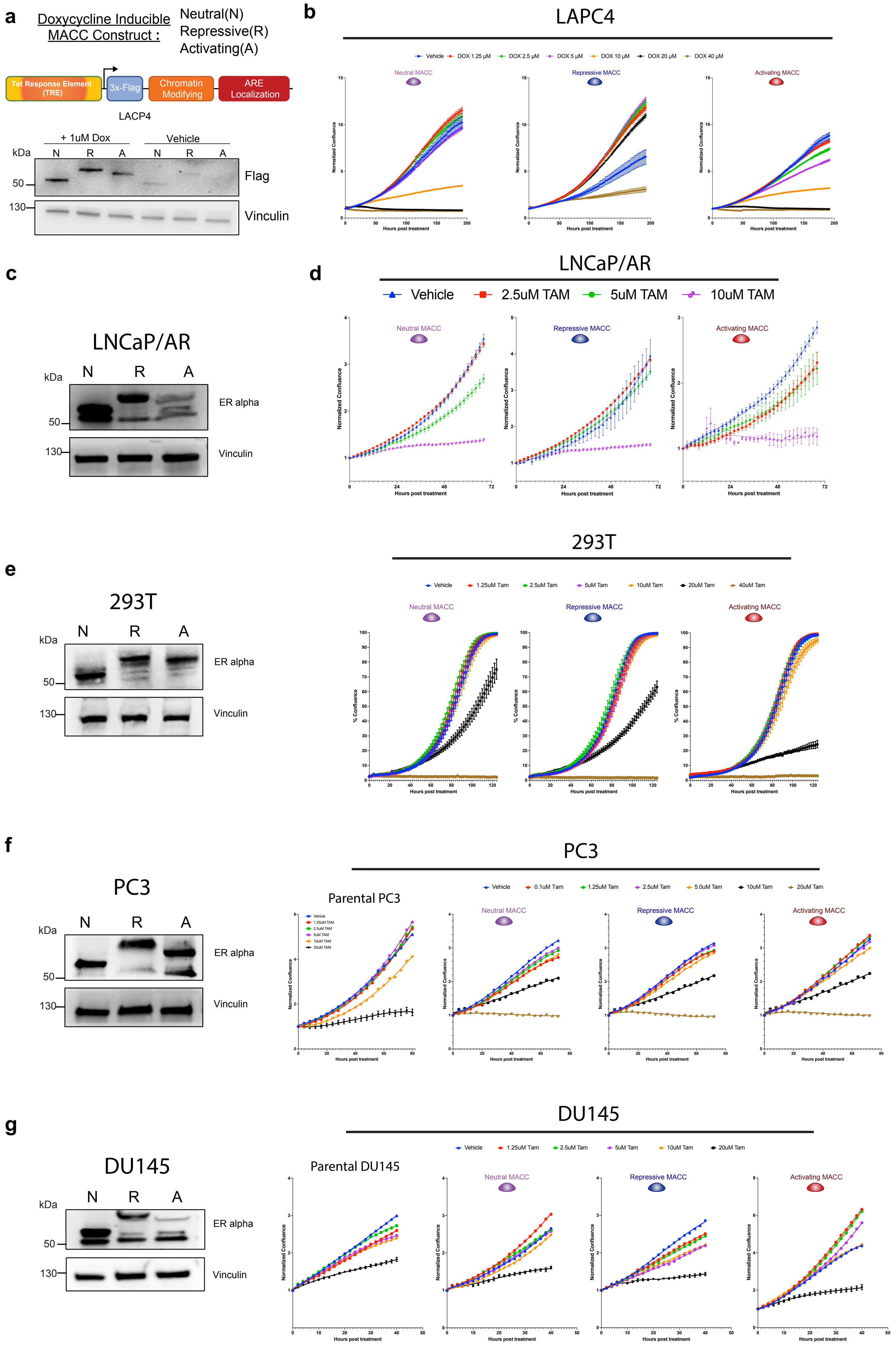
a
Doxycycline Inducible MACC Construct :
Neutral(N)
Repressive(R)
Activating(A)
Tet Response Element (TRE)
3x-Flag
Chromatin Modifying
ARE Localization
LACP4
+ 1uM Dox
Vehicle
kDa
N
R
A
N
R
A
50
130
Flag
Vinculin
b
LAPC4
Vehicle
DOX 1.25 μM
DOX 2.5 μM
DOX 5 μM
DOX 10 μM
DOX 20 μM
DOX 40 μM
Neutral MACC
Repressive MACC
Activating MACC
Normalized Confluence
Hours post treatment
c
LNCaP/AR
kDa
N
R
A
50
130
ER alpha
Vinculin
d
LNCaP/AR
Vehicle
2.5uM TAM
5uM TAM
10uM TAM
Neutral MACC
Repressive MACC
Activating MACC
Normalized Confluence
Hours post treatment
e
293T
kDa
N
R
A
50
130
ER alpha
Vinculin
293T
Vehicle
1.25uM Tam
2.5uM Tam
5uM Tam
10uM Tam
20uM Tam
40uM Tam
Neutral MACC
Repressive MACC
Activating MACC
% Confluence
Hours post treatment
f
PC3
kDa
N
R
A
50
130
ER alpha
Vinculin
PC3
Vehicle
0.1uM Tam
1.25uM Tam
2.5uM Tam
5.0uM Tam
10uM Tam
20uM Tam
Parental PC3
Neutral MACC
Repressive MACC
Activating MACC
Normalized Confluence
Hours post treatment
g
DU145
kDa
N
R
A
50
130
ER alpha
Vinculin
DU145
Vehicle
1.25uM Tam
2.5uM Tam
5uM Tam
10uM Tam
20uM Tam
Parental DU145
Neutral MACC
Repressive MACC
Activating MACC
Normalized Confluence
Hours post treatment

Supplementary Fig. 5 MACC phenotypic responses are restricted to cell lines of prostate lineage that express AR.

a, Schematic of doxycycline inducible MACC constructs, and expression of neutral (3X FLAG only), repressive (KRAB) and activating (VP64) constructs upon doxycycline induction in LAPC4 cells. The samples derive from the same experiment, but different gels for Flag and Vinculin were processed in parallel. **b,** Growth of LAPC4 MACC lines with vehicle or doxycycline treatment, n=3. **c-d,** Immunoblot and growth of LNCaP/AR MACC lines with vehicle or increasing doses of tamoxifen treatment, n=3. The samples derive from the same experiment, but different gels for ERa and Vinculin were processed in parallel. **e,** Immunoblot and growth of 293T MACC lines with vehicle or increasing doses of tamoxifen treatment, n=3. The samples derive from the same experiment, but different gels for ERa and Vinculin were processed in parallel. **f-g,** Immunoblot and growth of PC3 and DU145 (two AR-negative prostate cancer cell lines) MACC lines with vehicle or increasing doses of tamoxifen treatment, n=3. The samples derive from the same experiment, but different gels for ERa and Vinculin were processed in parallel. For experiments (**a–g**), the experiments were repeated 3 times (biological replicates), and a representative example is shown here. Source data are provided as a Source Data file.

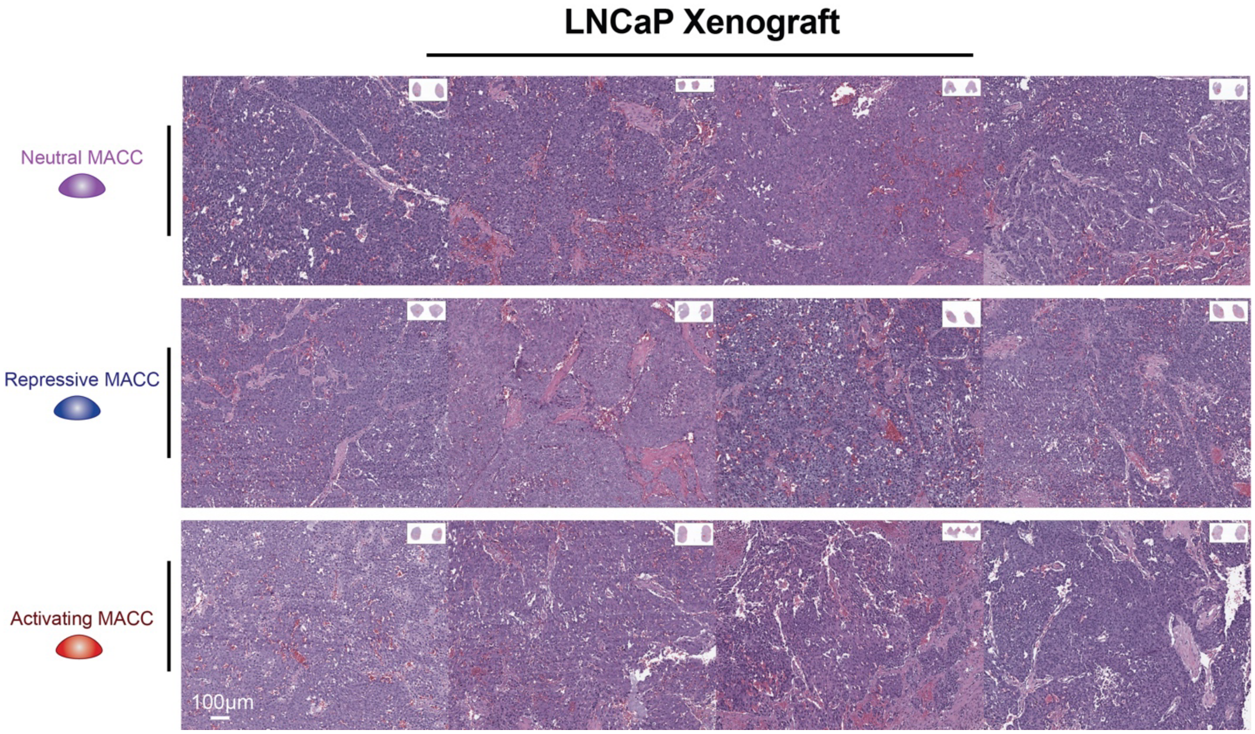

Supplementary Fig.6 Representative H&E staining of LNCaP xenograft tissue.
Images display the histological architecture for each sample. Scale bar represents 100 μm.

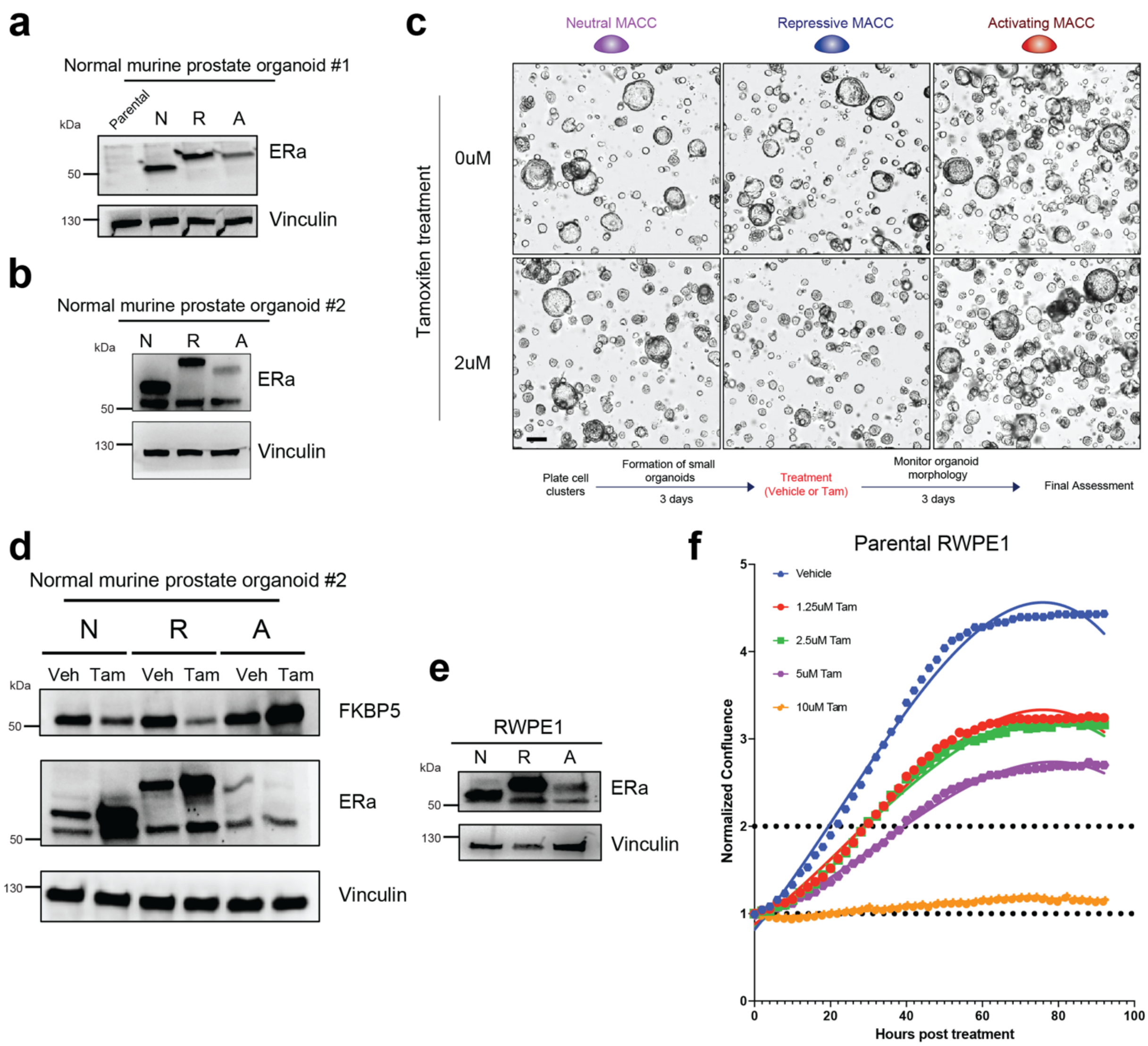

Supplementary Fig. 7 MACC effects in normal prostate cells.
a-b, Immunoblot of two independent genetically normal mouse prostate organoids with inducible MACC constructs. The samples derive from the same experiment, but different gels for ERa and Vinculin were processed in parallel. **c,** Brightfield images and growth of genetically normal mouse prostate organoids with inducible MACC constructs. Scale bar = 40 µm. **d,** Immunoblot of AR target FKBP5 in independent genetically normal mouse prostate organoids with inducible MACC constructs upon vehicle or 2uM tamoxifen treatment. The samples derive from the same experiment, but different gels for FKBP5, ERa and Vinculin were processed in parallel. **e,** Immunoblot of RWPE1 prostate lines with inducible MACC constructs. The samples derive from the same experiment, but different gels for ERa and Vinculin were processed in parallel. **f,** Growth of parental RWPE1 prostate lines with vehicle or increasing doses of tamoxifen, n=3. For experiments (**a**–**f**), the experiments were repeated 3 times (biological replicates), and a representative example is shown here. Source data are provided as a Source Data file.

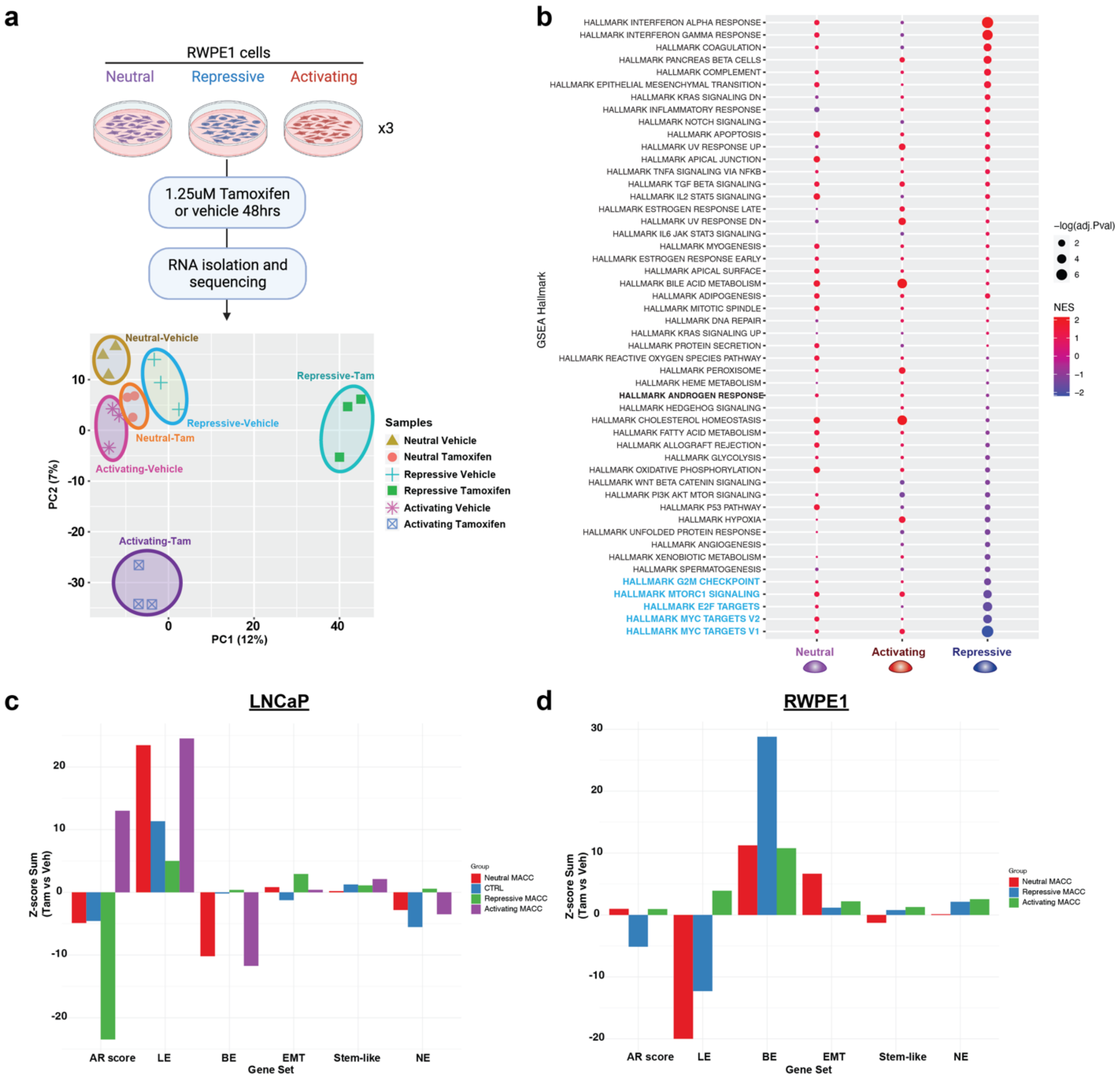

Supplementary Fig. 8 Transcriptional profiling, and pathway analysis of MACC lines.
a, Experimental plan for transcriptional profiling, and PCA of gene expression profiled from vehicle treated or tamoxifen induced RWPE1 MACC lines. **b,** Pathway analysis using GSEA Hallmarks of induced vs. vehicle RWPE1 MACC lines. **c-d,** Pathway analysis using AR score, luminal epithelia (LE), basal epithelial (BE), stem-like, neuroendocrine (NE), and epithelial-mesenchymal transition (EMT) of induced vs. vehicle MACC lines. **a,** Created with BioRender.com.

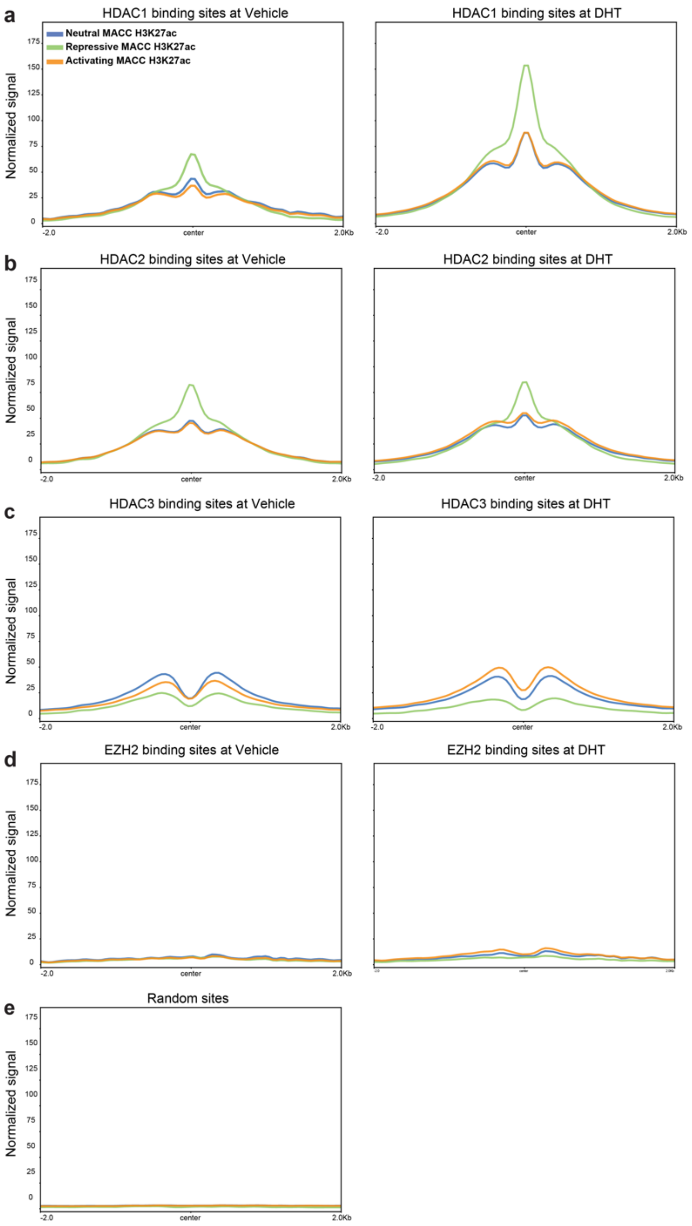

Supplementary Fig.9 MACC signals at HDAC1-3 and EZH2 binding peaks under vehicle-treated or DHT-treated conditions.

a-c, Signal of MACC H3K27ac at HDAC1-3 vehicle-treated or HDAC3 DHT-treated binding peaks. **d,** Signal of MACC H3K27ac at EZH2 vehicle-treated or HDAC3 DHT-treated binding peaks. **e,** Signal of MACC H3K27ac at random sites (hg19 genome).

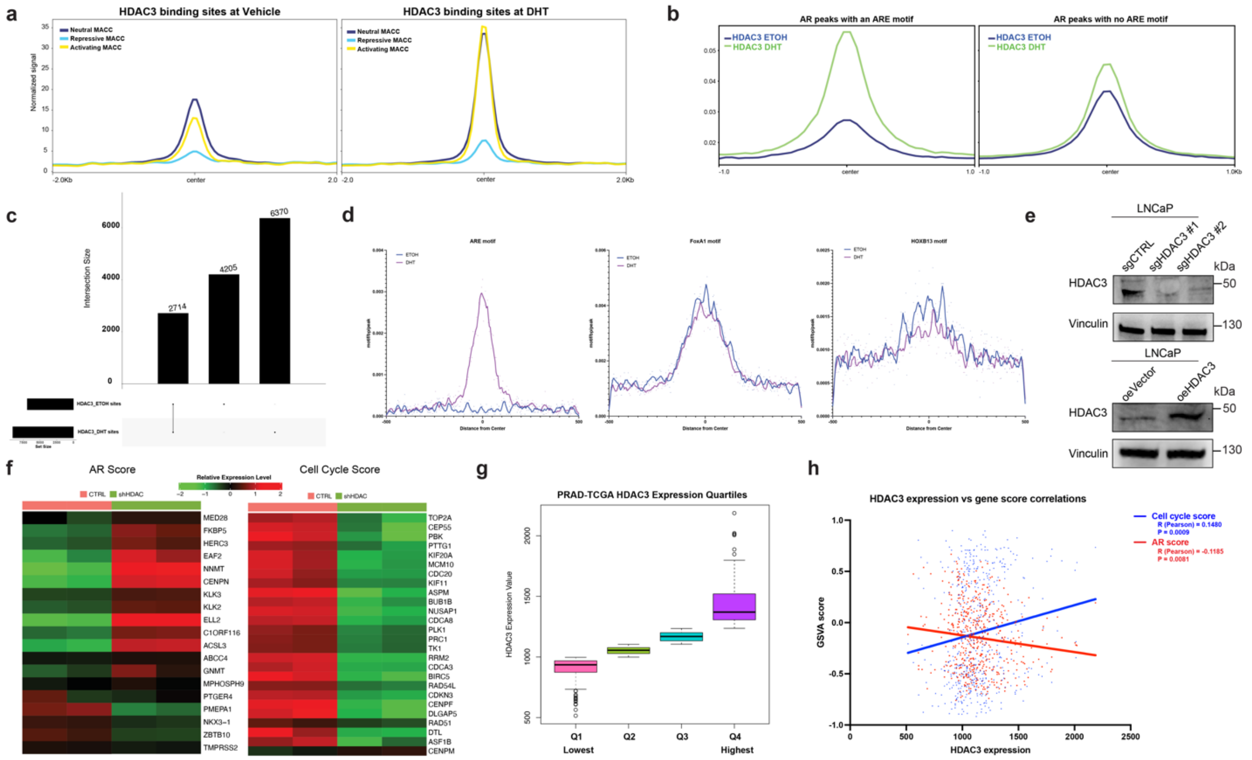

Supplementary Fig. 10 HDAC3's role on AR-mediated transcriptional regulation.

a, Comparison of MACC signals at all HDAC3 binding sites treated with vehicle (GSM717402) or DHT (GSM717403). **b,** Distribution of AR peak activities, distinguished by the presence or absence of AREs at HDAC3 binding sites. **c,** Upset plot illustrating the differential peak analysis across all HDAC3 binding sites treated with vehicle (GSM717402) and DHT (GSM717403). **d,** Histograms of the densities of ARE (left), FOXA1 (middle), and HOXB13 (right) motifs around the centers of HDAC3 binding peaks, based on ChIP-seq data from DHT-treated (purple line) and vehicle-treated (blue line) samples. **e,** Immunoblot of HDAC3 expression levels in LNCaP cells, comparing knockout and overexpression conditions. The samples derive from the same experiment, but different gels for HDAC3 and Vinculin were processed in parallel. **f,** Heatmap of the AR score and cell cycle score derived from LNCaP HDAC3-knockdown RNA-seq data (GSE153585). **g,** Boxplot of the distribution of HDAC3 expression levels, categorized into quartiles based on HDAC3 expression: Q1 (lowest expression, n=125), Q2 (n=122), Q3 (n=122), and Q4 (highest expression, n=122) within the TCGA cohort. **h,** Correlation plot of the relationship between HDAC3 expression and AR score, as well as cell cycle score in the TCGA cohort, with 'R' indicating the Pearson correlation coefficient, n=491. For (**e**) experiments were conducted at least three times with consistent results. Source data are provided as a Source Data file.

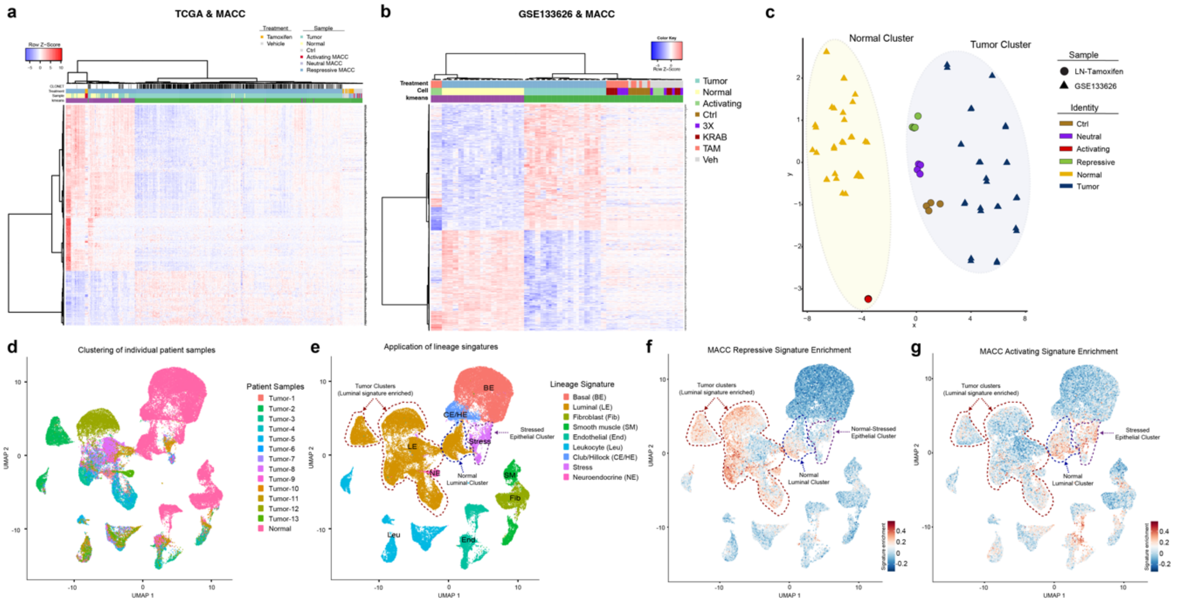

Supplementary Fig. 11 Unsupervised clustering of human PCa transcriptomes with MACC and enrichment of MACC signatures in tumor vs. normal prostate scRNA-seq samples.

a, Heatmap of unsupervised clustering of the transcriptomes of TCGA cohort, along with tamoxifen induced LNCaP MACC lines. **b,** Heatmap of unsupervised clustering of the transcriptomes of GSE133626 cohort, along with tamoxifen induced LNCaP MACC lines. **c,** Unsupervised clustering of the transcriptomes of human prostate cancer and normal samples from matched patient (GSE133626), along with tamoxifen induced LNCaP MACC lines. Induced activating MACC lines cluster with normal samples. **d,** Uniform Manifold Approximation and Projection (UMAP) view of single cells, color-coded by patient samples. **e,** UMAP view of single cells, color-coded by assigned cell type. **f-g,** UMAP view of single cells by expression level of MACC Repressive (**f**) and Activating (**g**) signatures.

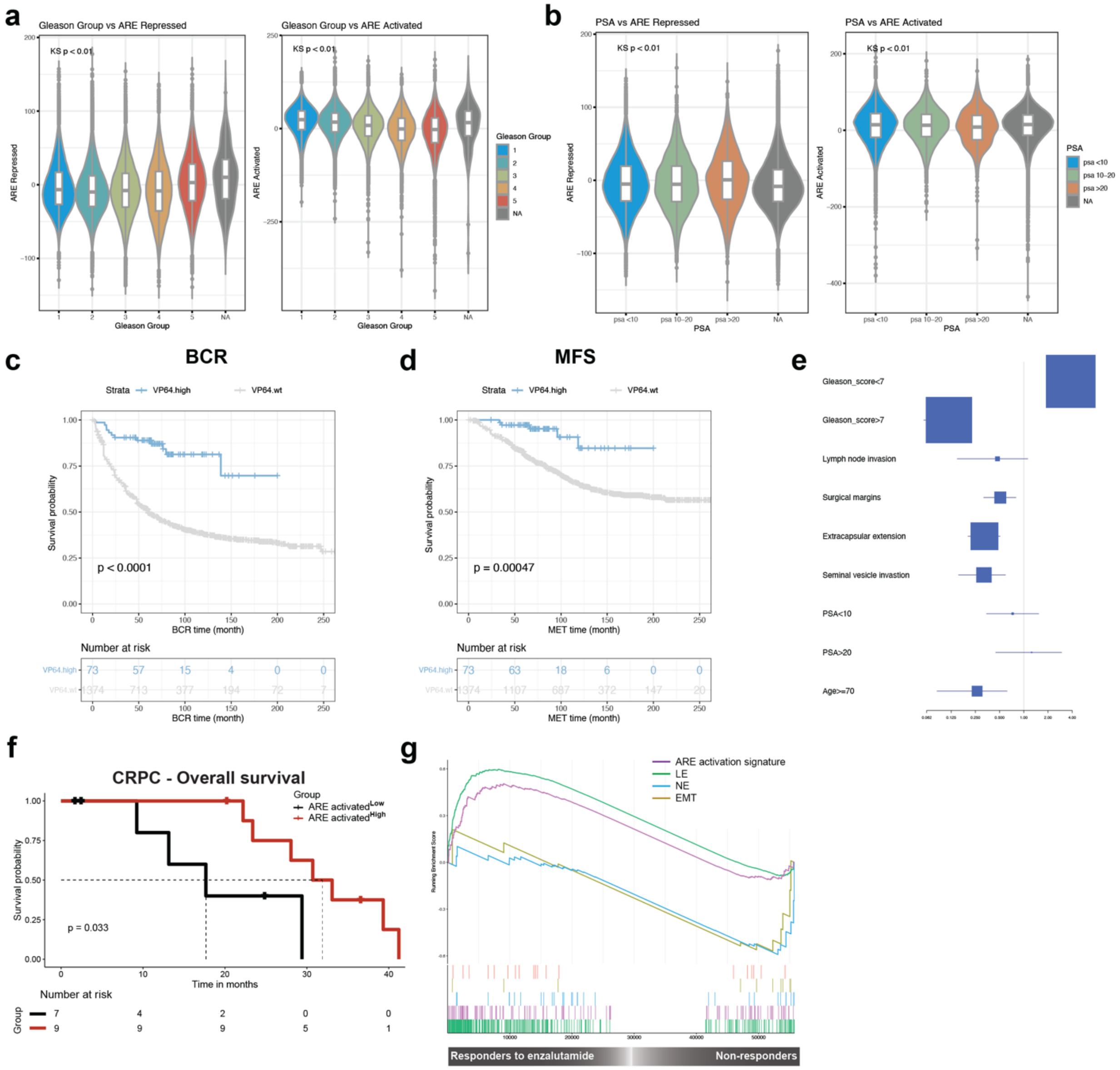

Supplementary Fig. 12 MACC transcriptional programs are clinically relevant.
a-b, Association of ARE activated and ARE repressed signatures from LNCaP MACC lines with Gleason group (**a**) and PSA level (**b**) in clinically localized prostate cancer, n=169,123. KS = Kruskal-Wallis. **c-d**, Kaplan Meier analysis of the biochemical recurrence (BCR) and metastasis-free survival (MFS) in a pooled retrospective cohort (n=1677), stratified by ARE activated (VP64) signature expression level. Time = months. **e,** Clinical and pathological associations of the ARE activated signatures from LNCaP MACC lines in the Decipher cohort via univariate analyses. **f,** Kaplan Meier analysis of overall survival in a cohort with metastatic castrate-resistant prostate cancer, stratified into half by ARE activated signature expression level. **g,** GSEA results for the ARE activation signature, LE, Stem-like, NE, and EMT phenotypes in non-responders and responders from a phase II trial of enzalutamide treatment in 36 men with metastatic CRPC.

Supplementary Table 1 ARE repression score with clinical characteristics in localized prostate cancer patient cohort.

ARE repression score with clinical characteristics in localized prostate cancer patient cohort					
	Q1: 42280 (25.00)	Q2: 42281 (25.00)	Q3: 42280 (25.00)	Q4: 42281 (25.00)	P Value
Median age at biopsy, yrs (range)	68.00[62.00 - 73.00]	68.00[62.00 - 73.00]	68.00[63.00 - 73.00]	69.00[63.00 - 74.00]	<0.001*
Gleason group, n (%)					<0.001*
1	9198 (21.76)	10718 (25.36)	10337 (24.46)	10712 (25.36)	
2	17990 (42.57)	18830 (44.55)	17708 (41.90)	15432 (36.53)	
3	9267 (21.93)	8197 (19.39)	8691 (20.57)	8688 (20.57)	
4	3475 (8.22)	2311 (5.47)	2565 (6.07)	3089 (7.31)	
5	2331 (5.52)	2212 (5.23)	2959 (7.00)	4319 (10.22)	
Median preoperative PSA value, n (range)	5.19[0.29 - 8.30]	5.10[0.28 - 8.10]	5.26[0.34 - 8.34]	5.17[0.38 - 8.42]	0.061
NCCN risk group after biopsy, n (%)					<0.001*
Low	6818 (24.04)	8145 (27.93)	7763 (27.77)	7818 (29.92)	
Int.Fav	6941 (24.48)	7528 (25.82)	7083 (25.34)	6374 (24.39)	
Int.Unfav	10450 (36.85)	10144 (34.79)	9426 (33.72)	7798 (29.84)	
High	3318 (11.70)	2662 (9.13)	2835 (10.14)	3091 (11.83)	
VeryHigh	832 (2.93)	682 (2.34)	843 (3.02)	1048 (4.01)	
Clinical stage, n (%)					<0.001*
T1	23739 (83.97)	24894 (85.42)	23692 (84.96)	22141 (85.14)	
T2	4217 (14.92)	3987 (13.68)	3892 (13.96)	3517 (13.52)	
T3	300 (1.06)	253 (0.87)	293 (1.05)	311 (1.20)	
T4	16 (0.06)	9 (0.03)	10 (0.04)	37 (0.14)	
Lymph node involvement, n (%)					<0.001*
No	41865 (99.02)	41842 (98.96)	41678 (98.58)	41322 (97.74)	
Yes	413 (0.98)	438 (1.04)	601 (1.42)	955 (2.26)	
Percentage of positive biopsy cores (range)	0.33[0.19 - 0.50]	0.33[0.17 - 0.50]	0.33[0.17 - 0.50]	0.25[0.17 - 0.50]	<0.001*
ARE repression score expression quartile, n (%)					<0.001*
Q1	42280 (100.00)	0 (0.00)	0 (0.00)	0 (0.00)	
Q2	0 (0.00)	42281 (100.00)	0 (0.00)	0 (0.00)	
Q3	0 (0.00)	0 (0.00)	42280 (100.00)	0 (0.00)	
Q4	0 (0.00)	0 (0.00)	0 (0.00)	42281 (100.00)	
ARE activation score expression quartile, n (%)					<0.001*
Q1	6836 (16.17)	7160 (16.93)	10394 (24.58)	17890 (42.31)	
Q2	10388 (24.57)	11016 (26.05)	11604 (27.45)	9272 (21.93)	
Q3	12087 (28.59)	12187 (28.82)	10422 (24.65)	7584 (17.94)	
Q4	12969 (30.67)	11918 (28.19)	9860 (23.32)	7534 (17.82)	

PSA, prostate-specific antigen; NCCN, National Comprehensive Cancer Network; Int.Fav, Favorable Intermediate risk; Int.Unfav, Unfavorable Intermediate risk; *, statistical significance. Statistical significance was determined using the Chi-squared test for categorical variables and the Kruskal-Wallis test for continuous variables. n = number of patients.

Supplementary Table 2 Univariable and multivariable Cox regression analyses of ARE repression score.

Variable Name	Univariable Hazard Ratio (95% CI)	P-value	Multivariable Hazard Ratio (95% CI)	P-value
Age	0.97 (0.94-1.00)	0.030*	0.96 (0.94-0.99)	0.010*
Prostate Specific Antigen between 10 to 20 ng/mL	1.01 (0.61-1.69)	0.961	0.80 (0.46-1.39)	0.429
Prostate Specific Antigen > 20 ng/mL	1.39 (0.73-2.63)	0.318	0.95 (0.48-1.89)	0.89
Gleason Score = 7	7.13 (0.97-52.31)	0.053	5.06 (0.68-37.75)	0.114
Gleason Score 8-10	22.36 (3.05-163.73)	0.002*	12.98 (1.72-97.83)	0.013*
Surgical Margin Status (SM)	1.54 (0.95-2.48)	0.08	1.36 (0.84-2.21)	0.207
Extracapsular Extension (ECE)	3.40 (2.02-5.70)	<0.001*	2.69 (1.46-4.98)	0.002*
Seminal Vesicle Invasion (SVI)	3.05 (1.97-4.71)	<0.001*	1.77 (1.09-2.88)	0.022*
Lymph Node Involvement (LNI)	4.71 (2.74-8.08)	<0.001*	2.37 (1.30-4.32)	0.005*
ARE repression score	1.07 (1.01-1.14)	0.031*	1.04 (0.98-1.12)	0.216

CI: Confidence interval; *, statistical significance. Statistical significance was determined using Cox proportional hazards regression models.

Supplementary Table 3 ARE activation score with clinical characteristics in localized prostate cancer patient cohort.

ARE activation score with clinical characteristics in localized prostate cancer patient cohort					
	Q1: 42280 (25.00)	Q2: 42281 (25.00)	Q3: 42280 (25.00)	Q4: 42281 (25.00)	P Value
Median age at biopsy, yrs (range)	69.00[64.00 - 74.00]	69.00[63.00 - 74.00]	68.00[62.00 - 73.00]	67.00[61.00 - 72.00]	<0.001*
Gleason group, n (%)					<0.001*
1	7497 (17.74)	9093 (21.52)	11261 (26.65)	13114 (31.04)	
2	15127 (35.80)	18104 (42.84)	18588 (43.98)	18142 (42.94)	
3	10299 (24.37)	9380 (22.19)	8148 (19.28)	7016 (16.61)	
4	4451 (10.53)	2928 (6.93)	2160 (5.11)	1901 (4.50)	
5	4880 (11.55)	2757 (6.52)	2106 (4.98)	2077 (4.92)	
Median preoperative PSA value, n (range)	5.08[0.30 - 8.30]	5.30[0.39 - 8.50]	5.30[0.34 - 8.40]	5.10[0.29 - 8.10]	0.001*
NCCN risk group after biopsy, n (%)					<0.001*
Low	5467 (20.74)	6690 (23.35)	8494 (28.85)	9893 (36.44)	
Int.Fav	5747 (21.81)	7026 (24.52)	7840 (26.63)	7313 (26.93)	
Int.Unfav	9054 (34.35)	10850 (37.87)	10094 (34.28)	7820 (28.80)	
High	4297 (16.30)	3242 (11.32)	2543 (8.64)	1824 (6.72)	
VeryHigh	1790 (6.79)	841 (2.94)	471 (1.60)	302 (1.11)	
Clinical stage, n (%)					<0.001*
T1	21412 (82.19)	24124 (84.41)	25168 (85.44)	23762 (87.30)	
T2	4091 (15.70)	4124 (14.43)	4084 (13.86)	3313 (12.17)	
T3	513 (1.97)	316 (1.11)	194 (0.66)	134 (0.49)	
T4	35 (0.13)	17 (0.06)	10 (0.03)	10 (0.04)	
Lymph node involvement, n (%)					<0.001*
No	41411 (97.95)	41703 (98.64)	41826 (98.93)	41767 (98.79)	
Yes	866 (2.05)	575 (1.36)	454 (1.07)	512 (1.21)	
Percentage of positive biopsy cores (range)	0.33[0.17 - 0.56]	0.33[0.17 - 0.50]	0.33[0.17 - 0.50]	0.27[0.17 - 0.44]	<0.001*
ARE repression score expression quartile, n (%)					<0.001*
Q1	6836 (16.17)	10388 (24.57)	12087 (28.59)	12969 (30.67)	
Q2	7160 (16.93)	11016 (26.05)	12187 (28.82)	11918 (28.19)	
Q3	10394 (24.58)	11604 (27.45)	10422 (24.65)	9860 (23.32)	
Q4	17890 (42.31)	9272 (21.93)	7584 (17.94)	7534 (17.82)	
ARE activation score expression quartile, n (%)					<0.001*
Q1	42280 (100.00)	0 (0.00)	0 (0.00)	0 (0.00)	
Q2	0 (0.00)	42281 (100.00)	0 (0.00)	0 (0.00)	
Q3	0 (0.00)	0 (0.00)	42280 (100.00)	0 (0.00)	
Q4	0 (0.00)	0 (0.00)	0 (0.00)	42281 (100.00)	

PSA, prostate-specific antigen; NCCN, National Comprehensive Cancer Network; Int.Fav, Favorable Intermediate risk; Int.Unfav, Unfavorable Intermediate risk; *, statistical significance. Statistical significance was determined using the Chi-squared test for categorical variables and the Kruskal-Wallis test for continuous variables. n = number of patients.

Supplementary Table 4 Univariable and multivariable Cox regression analyses of ARE activation score.

Variable Name	Univariable Hazard Ratio (95% CI)	P-value	Multivariable Hazard Ratio (95% CI)	P-value
Age	0.97 (0.94-1.00)	0.030*	0.96 (0.94-0.99)	0.005*
Prostate Specific Antigen between 10 to 20 ng/mL	1.01 (0.61-1.69)	0.961	0.79 (0.46-1.36)	0.396
Prostate Specific Antigen > 20 ng/mL	1.39 (0.73-2.63)	0.318	0.94 (0.48-1.83)	0.851
Gleason Score = 7	7.13 (0.97-52.31)	0.053	5.02 (0.67-37.54)	0.116
Gleason Score 8-10	22.36 (3.05-163.73)	0.002*	12.94 (1.71-97.84)	0.013*
Surgical Margin Status (SM)	1.54 (0.95-2.48)	0.08	1.24 (0.78-1.99)	0.363
Extracapsular Extension (ECE)	3.40 (2.02-5.70)	<0.001*	2.25 (1.27-4.01)	0.006*
Seminal Vesicle Invasion (SVI)	3.05 (1.97-4.71)	<0.001*	1.84 (1.14-2.97)	0.012*
Lymph Node Involvement (LNI)	4.71 (2.74-8.08)	<0.001*	2.69 (1.52-4.77)	<0.001*
ARE activation score	0.91 (0.87-0.96)	<0.001*	0.91 (0.86-0.96)	<0.001*

CI: Confidence interval; *, statistical significance. Statistical significance was determined using Cox proportional hazards regression models.

Supplementary Table 5 Oligos or DNA sequences used in this study.

Oligos or DNA sequences	Forward primer	Reverse primer
Primers for amplification of 3xFlag	AAAGCTAGCATGGACTATAAGGACCACGACGGAGA	AAAGCTAGCGGTACCGGATCCACCGCCGGACCC
Primers for amplification of KRAB	AAAGCTAGCATGGACTATAAGGACCACGACGGAGA	AAAGCTAGCGCCGCTGCCGCCTGAGCCAC
Primers for amplification of VP64	AAAGCTAGCATGGACTACAAAGACCATGACGGTGA	AAAGCTAGCTAACATATCGAGATCGAAATCGTCCAGAGC
Sequence of AR DNA binding domain (DBD) used in MACC	TTGGAGACTGCCAGGGACCATGTTTTGCCCATTGACTATTACTTTCCACCCCAGAAGACCTGCCTGATCTGTGGAGATGAAGCTTCTGGGTGTCACTATGGAGCTCTCACATGTGGAAGCTGCAAGGTCTTCTTCAAAAGAGCCGCTGAAGGGAAACAGAAGTACCTGTGCGCCAGCAGAAATGATTGCACTATTGATAAATTCCGAAGGAAAAATTGTCCATCTTGTCGTCTTCGGAAATGTTATGAAGCAGGGATGACTCTGGGAGCCCGGAAGCTGAAGAAACTT	

Supplementary Table 6 Antibodies used in this study.

Target	Vendor and Catalog Number	RRID	Usage	Dilution
Vinculin	Abcam Cat# ab129002	AB_11144129	WB	1:10000 for WB
FKBP5	Cell Signaling Technology Cat# 12210	AB_2797846	WB	1:1000 for WB
ERalpha (ERa)	Santa Cruz Biotechnology Cat# sc-8002	AB_627558	WB; IF;ChIP-seq	1:500 for WB; 1:250 for IF; 5ug for ChIP
FLAG	Sigma-Aldrich Cat# F3165	AB_259529	WB; IF	1:1000 for WB; 1: 1000 for IF
AR	Abcam Cat# ab108341	AB_10865716	WB	1:1000 for WB
Cytokeratin 5 (CK5)	BioLegend Cat# 905901	AB_2565054	IF	1:250 for IF
Cytokeratin 8 (CK8)	Abcam Cat# ab53280	AB_869901	IF	1:250 for IF
HDAC3	Cell Signaling Technology Cat# 3949T	AB_2118371	WB	1:1000 for WB
H3K27ac	Abcam Cat# ab4729	AB_2118291	ChIP; ChIP-seq	5ug for ChIP
WB - Western Blot; IF - Immunofluorescence; ChIP - Chromatin Immunoprecipitation.				
